# The Influence of Various Crosslinking Conditions of EDC/NHS on the Properties of Fish Collagen Film

**DOI:** 10.3390/md22050194

**Published:** 2024-04-25

**Authors:** Alina Sionkowska, Karolina Kulka-Kamińska, Patrycja Brudzyńska, Katarzyna Lewandowska, Łukasz Piwowarski

**Affiliations:** 1 Department of Biomaterials and Cosmetic Chemistry, Nicolaus Copernicus University in Torun, Gagarina 7, 87-100 Torun, Poland; kkulka@doktorant.umk.pl (K.K.-K.); patrycja.brudzynska@umk.pl (P.B.); reol@umk.pl (K.L.); 2 SanColl Sp. z o. o., Juliusza Słowackiego 24, 35-060 Rzeszów, Poland; aktivpiwowarski@gmail.com

**Keywords:** fish collagen, EDC, NHS, crosslinking, biopolymer film

## Abstract

The process of crosslinking improves the physicochemical properties of biopolymer-based composites, making them valuable for biomedical applications. EDC/NHS-crosslinked collagen materials have a significant potential for tissue engineering applications, due to their enhanced properties and biocompatibility. Chemical crosslinking of samples can be carried out in several ways, which is crucial and has a direct effect on the final properties of the obtained material. In this study, the effect of crosslinking conditions on the properties of collagen films using EDC and NHS was investigated. Studies included FTIR spectroscopy, AFM, swelling and degradation tests, mechanical testing and contact angle measurements. Evaluation of prepared collagen films indicated that both crosslinking agents and crosslinking conditions influenced film properties. Notable alternations were observed in the infrared spectrum of the sample, to which EDC was added directly to the fish collagen solution. The same sample indicated the lowest Young modulus, tensile strength and breaking force parameters and the highest elongation at break. All samples reached the maximum swelling degree two hours after immersion in PBS solution; however, the immersion-crosslinked samples exhibited a significantly lower degree of swelling and were highly durable. The highest roughness was observed for the collagen film crosslinked with EDC, whereas the lowest was observed for the specimen crosslinked with EDC with NHS addition. The crosslinking agents increased the surface roughness of the collagen film, except for the sample modified with the addition of EDC and NHS mixture. All films were characterized by hydrophilic character. The films’ modification resulted in a decrease in their hydrophilicity and wettability. Our research allows for a comparison of proposed EDC/NHS crosslinking conditions and their influence on the physicochemical properties of fish collagen thin films. EDC and NHS are promising crosslinking agents for the modification of fish collagen used in biomedical applications.

## 1. Introduction

One of the most widely used protein biopolymers in medicine, cosmetics, pharmaceuticals and nutraceuticals is collagen [1]. It is a structural protein found in humans and animals and is an important component of the extracellular matrix (ECM), accounting for up to 25–30% of the protein content of organisms. This fibrillar protein provides elasticity and strength to organ tissues and is found primarily in fibrous connective tissues, including fascia, ligaments, vascular appendages, joint capsules, cartilage and dermis [2]. Collagen has a triple helix structure, with the three parallel polypeptide strands coiled left-handed [3,4]. There are 28 different types of collagen, which show a considerable diversity in both function and structure. The most common type of collagen in humans is type I collagen, which is found mostly in the skin, bones and tendons [5,6]. The source of collagen is of great importance in terms of physicochemical properties, thermal stability and viscosity of the resulting product due to differences in amino acid composition [7]. Animal products, including rat tails and pig and bovine hides, are the main sources of collagen. Pork and beef products have been the mainstay of industrial collagen production for many years, but their use has declined in recent years. Consumer choices and dietary habits, as well as the risk of zoonotic disease transmission (TSE, BSE, FMD) have contributed to the decline in the use of collagen from these sources [1]. An alternative source of collagen is marine organisms [1,2,7,8,9,10]. The oceans are among the richest and most diverse ecosystems on Earth. They are a valuable source of natural food and active ingredients. For example, fish skin and bones alone are a rich source of collagen and calcium phosphates, respectively, in addition to other marine organisms [11]. Unfortunately, up to 80% of marine organisms are inedible [12]. Food processing waste is a major source of pollution and a social and economic problem. For these reasons, it is desirable to process the residues to isolate other valuable components. The European Commission has adopted the “Blue Growth” strategy to promote sustainable development in the marine sector [8]. Marine collagen has been approved by the Food and Drug Administration (FDA) as safe for use without the risk of disease transmission [12]. It is a biocompatible product with excellent biodegradability, antioxidant properties and also acts as a humectant with the ability to hydrate the skin and delay the aging process [1,3]. Other advantages of this macromolecule obtained from marine sources are its high water absorption capacity and low immunogenicity [13], and what is more, several studies confirm its wound healing potential [9]. Collagen can be extracted from jellyfish, sharks, starfish, sponges, octopuses, squids, sea cucumbers and fish [1,2,8,9]. The advantages of this type of collagen source are its low cost, its low molecular weight which is suitable for human absorption and its high collagen content [14]. It has been reported that fish collagen has similar properties to mammalian collagen; this includes its molecular structure and biochemical properties [13]. Fish collagen can be easily extracted and the substrates in this process can be skin, scales, bones, skull and swim bladder [13,15]. The fish species frequently used for collagen production are *Rachycentron canadum*, *Spottles smooth hound*, *Sardinella fimbriata*, *Scomber japonicus*, *Gadus morhua*, *Alaska pollock*, *Oreochromis niloticus*, and many more [13,14]. Depending on the species of origin, there may be slight differences in collagen characteristics, especially in denaturation temperature; these variations are mainly due to differences in the hydroxyproline content of the polymer chain [7]. One example is silver carp (*Hypopthalmichthys molitrix*) collagen, which has higher heat resistance than collagen from other species [16]. Collagen extraction from marine sources can be performed by conventional (chemical hydrolysis, salt solubilization) and novel methods (ultrasound-assisted extraction, microwave-assisted extraction, enzymatic hydrolysis) [12,17]. Generally, collagen extraction methods consist of the following steps: tissue separation and purification (1), tissue fragmentation (2), elimination of non-collagen parts and demineralization (3), extraction (4) and precipitation (5) [1,12,13]. Depending on which part of the fish the collagen is extracted from, different extraction process conditions are required, including temperature and time [14].

Despite its many benefits, marine collagen has some drawbacks such as low denaturation temperatures, low mechanical properties and rapid biodegradation. To improve the mechanical properties of collagen specimens, a crosslinking process is carried out [18]. Chemical, enzymatic and physical crosslinking methods can be distinguished. Enzymatic crosslinking, which occurs naturally in tissues, is extremely complex and difficult to reproduce under laboratory conditions, also taking into account the differences in the process in individual tissues [19]. Therefore, chemical and physical collagen modification methods are more widely used in many applications, depending on the active substance release time required [20]. The popular synthetic crosslinking agents are aldehydes (GTA, formaldehyde), ethylene glycol dimethyacrylate and epichlorohydrin. Unfortunately, if the cleaning process is poorly performed, the toxicity is the reason for the poor biocompatibility of samples containing these chemicals [20]. Carbodiimide chemistry has been used for crosslinking more frequently in recent years [19]. It improves the biochemical characteristics of materials without undesirable effects, such as toxic degradation products. EDC (1-ethyl-(3-dimethylaminopropyl)carbodiimide) is a zero-length crosslinker capable of forming an isopeptide bond between carboxyl and amine reactive groups and is not incorporated into the final product [21,22,23]. What is more, urea, which is a by-product of the crosslinking reaction, can be easily removed by rinsing. EDC is very often used in a mixture with NHS (N-hydroxysuccinimide), which occurs as an EDC hydrolysis suppressor agent. This reaction mixture induces the amide bond formation (by activating the side-chain carboxylic groups of glutamic and aspartic acid residues), followed by the aminolysis of the o-isoacylurea intermediates by the ϵ-amino groups of (hydroxy-)lysine residues, forming intra- and interhelical cross-connections [24,25]. The EDC/NHS molar ratio against the carboxylic groups is significant in terms of the coupling reaction rate. The advantages of this crosslinking method are the water solubility of EDC and its mild effect without the risk of protein denaturation, but the drawbacks include the possible stiffening of the tissue [25]. These two crosslinkers are less toxic when compared to glutardehyde and can be applied in biomaterials [26]. EDC/NHS-crosslinked collagen materials have a significant potential for tissue engineering applications, due to their excellent mechanical properties and biocompatibility [27]. According to research, EDC and NHS increase the mechanical strength of scaffolds made of type I collagen with minimal toxic effects on chondrocytes, which can be reduced through the cleaning process. The efficacy of collagen crosslinking with EDC/NHS can be increased by the application of other methods [26]. Moreover, several studies have shown that such composites may also constitute valuable biomaterial with the prospect of application in bone tissue engineering [23,28]. The study conducted by Diogo et al. indicated that the scaffold obtained from the marine collagen originating from shark skin mixed with the calcium phosphates extracted from the teeth of two different shark species, which were crosslinked with EDC/NHS, can support attachment and proliferation of osteoblast-like cells and thus, can act as a potential 3D material for application in hard tissue as a promising structure for bone regeneration [11].

Chemical crosslinking of samples can be carried out in a number of ways, including adding the crosslinking agent directly to the solution or immersing the sample in the crosslinking agent solution. The way in which the specimen is prepared is important and has a direct effect on the properties of the sample. In this study, the effect of the way a sample is prepared on the properties of collagen films using EDC was investigated. The impact of the presence of NHS in the crosslinking mixture was also examined. Studies included infrared spectroscopy, atomic force microscopy, swelling and degradation tests, mechanical properties evaluation, as well as contact angle measurements. Applying chemicals such as EDC, NHS and their mixture to collagen films has already been performed, whereas to the best of our knowledge, the comparison of the physicochemical properties of fish collagen films crosslinked by the direct addition of EDC and EDC/NHS and immersion in EDC and EDC/NHS solution is a novelty.

## 2. Results

### 2.1. Fourier Transform Infrared Spectroscopy

**Figure 1:**
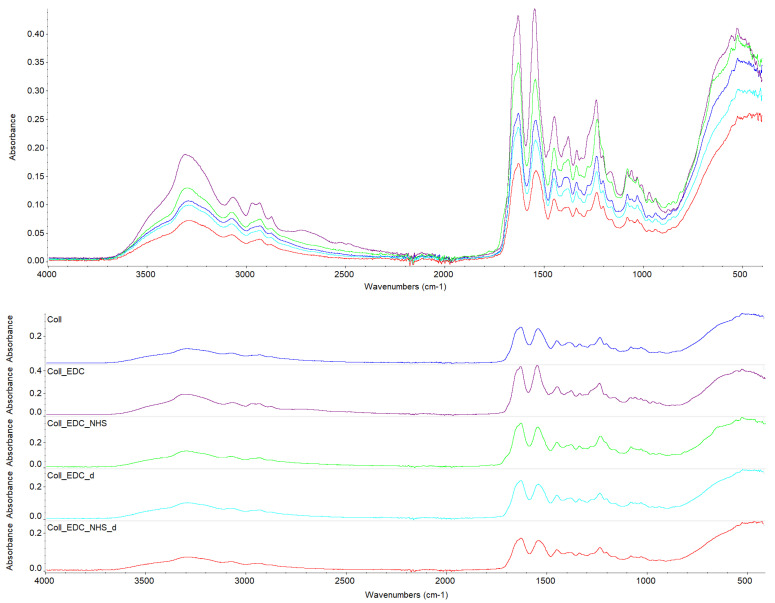
The IR spectra of collagen films non-crosslinked and crosslinked with EDC or EDC/NHS. Spectra are presented in stacks (above) and overview (below).

FTIR spectra of unmodified and crosslinked with EDC or EDC/NHS collagen films are shown in Figure 1. Table 1 presents the samples’ wavenumbers in characteristic bands. The pure collagen film’s infrared spectrum presents main bands as follows: amide A, amide B and amides I–III. The biggest changes are observed in the FTIR spectrum of film Coll_EDC, where EDC was added directly to the fish collagen solution; for this sample, there are shifts in the amide II band region, towards higher wavenumbers. Significant changes are also observed in the region of the COO^−^ vibration and in the amide III region for the Coll_EDC, as well as Coll_EDC/NHS samples. In the spectrum registered for the sample Coll_EDC, the peak of amide III has the highest intensity while for the Coll_EDC/NHS, the peak is shifted with the change in spectra shape. All these alterations may indicate the activation of collagen carboxyl groups in the formation of new bonds, consistent with the action mechanism of EDC [24]. What is more, in Coll_EDC spectra, there is an additional band at a wavenumber of 1060 cm^−1^. This large number of variations in the spectrum for the EDC-modified sample may be due to the presence of the residual compound, especially as a rather high concentration was used. There are no significant changes in amide bands for the rest of the samples, besides the variations in intensity.

### 2.2. Mechanical Testing

**Figure 2:**
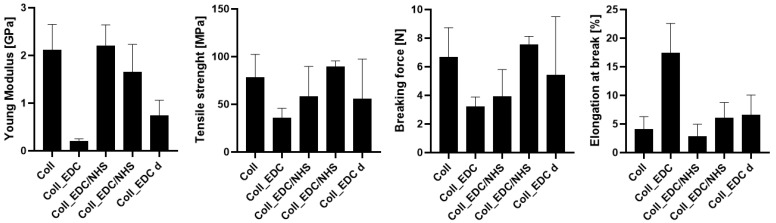
Young modulus, tensile strength, breaking force and elongation at break (from the left) for unmodified and crosslinked collagen films.

It can be noticed that crosslinking agents such as EDC and NHS had an impact on the mechanical properties of the collagen film obtained from 1% solution. The results of mechanical testing are presented in Figure 2. In particular, significantly different results were observed for samples with EDC addition. Films made of collagen with EDC indicated the lowest Young modulus, tensile strength and breaking force parameters among all tested specimens as well as the highest elongation at break; thus, this type of film was the most flexible. There were no significant differences among samples crosslinked with EDC/NHS using two methods in tensile strength parameter and among samples obtained by dipping in elongation at break parameter. Furthermore, in comparison to native collagen samples, Young modulus was similar for samples crosslinked with EDC/NHS, while it was lower for samples obtained by the dipping method. Only for the sample crosslinked with EDC by the dipping method was the tensile strength slightly higher compared to the collagen film, whereas only for the sample crosslinked with EDC/NHS was the elongation at break lower than for pure collagen samples. 

### 2.3. Swelling and Degradation Properties

**Figure 3:**
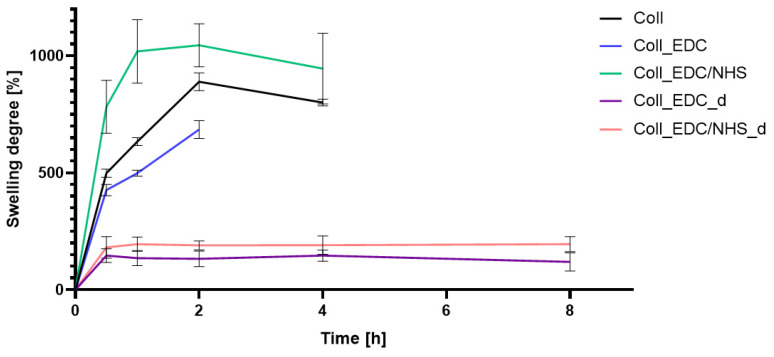
Swelling degree of collagen films non-crosslinked and crosslinked with EDC or EDC/NHS.

The samples achieved maximum swelling approximately two hours after the start of the analysis. The pure collagen film swelled to 889% and disintegrated after four hours. The Coll_EDC/NHS samples exhibited the highest degree of swelling, which also disintegrated after four hours. The Coll_EDC film disintegrated the fastest, after only two hours. The immersion-crosslinked samples showed a low degree of swelling but were highly durable. After 2 days, a decrease in the weight of the samples was observed, indicating degradation processes. Samples crosslinked with EDC alone showed a lower swelling degree compared to samples with the addition of NHS. Long-term and short-term results of swelling properties are presented as averages with standard deviation in Table 2 and Figure 3. 

### 2.4. Atomic Force Microscopy

Atomic force microscopy was used to investigate the surface morphology of obtained samples. Roughness values are shown in Table 3, while AFM images of collagen films with crosslinkers are gathered and shown in Table 4. Differences in roughness parameters between collagen films was noticeable after the crosslinking process, so it can be deduced that the crosslinkers modified the collagen films’ surface morphology. The highest values were obtained for the Rq parameter; thus, the highest roughness was observed for collagen films crosslinked with EDC, whereas the lowest was observed for the specimen crosslinked with EDC with NHS addition (58.32 and 13.31, respectively). Only for the latter is the surface roughness lower than for the pure collagen film, while for the three remaining samples, the crosslinking agents increased the surface roughness of the collagen films. 

### 2.5. Contact Angle and Surface Energy

The results of contact angle measurements and surface free energies are summarized in Table 5. Due to the significant distortion of the sample Coll_EDC/NHS_d, performing this analysis was not possible. All analyzed samples have hydrophilic characteristics. Crosslinking agent addition significantly influences the samples’ surface properties. The films’ modification resulted in a decrease in their hydrophilicity and a decrease in the polar surface energy component. The analysis revealed that the most notable changes occurred for the Coll_EDC sample. The data obtained indicate a decrease in the wettability of the crosslinked samples.

## 3. Discussion

The investigation of collagen films crosslinked with EDC and NHS indicated that both crosslinking agents, their amount and crosslinking conditions influenced the mechanical properties, swelling degree, surface roughness, wettability and surface free energy of the prepared materials. FTIR analysis revealed that significant alternations were observed in the spectrum of the sample to which EDC was added directly to the fish collagen solution. All observed changes may be related to the activation of collagen carboxyl groups in the formation of new bonds, consistent with the action mechanism of EDC. As mentioned, in Coll_EDC spectra, an additional band at the wavenumber of 1060 cm^−1^ was found, which is related to the presence of ester groups. Peaks in the regions 1277–1185 cm^−1^ and 1160–1050 cm^−1^ are due to the C-O ester groups and are correlated with asymmetrical and symmetrical stretching frequencies [29]. However, for the remaining samples, no significant changes in FTIR spectra were observed, besides the variations in intensity. Wang et al., in a study of different collagen crosslinking methods for dentin applications, suggested that EDC/NHS coupling caused very small, almost unnoticeable changes in the FTIR spectra [30]. In the study conducted by Nair et al., FTIR spectra demonstrated little alternations to the triple helical conformation after crosslinking collagen films with a mixture of EDC and NHS [27]. Films made of collagen crosslinked with EDC indicated the lowest Young modulus, tensile strength and breaking force parameters among all samples and the highest elongation at break, and thus were characterized by greater flexibility. According to Everaerts et al.’s research, collagen materials modified with EDC/NHS presented an increase in strain values at break; moreover, materials with blocked amine groups indicated the highest stress at break and reduced strain at a given stress in comparison to unmodified collagen patches. In general, this study indicated that additional ester bonds influence a material’s mechanical properties [29]. Zhang et al.’s research, which concerned fish swim bladder membranes that are rich in collagen crosslinked with EDC/NHS, showed that treated materials presented higher tensile stress and lower breaking elongation [31]. Furthermore, Nair et al.’s study revealed that tensile modulus increased for EDC/NHS-crosslinked collagen films [27]. Also, Grover et al. observed increased strength, stress at failure and Young modulus after treatment with EDAC/NHS reagents in comparison to native collagen films [32]. Similar observations were made by Wang et al., where EDC/NHS was added directly to the collagen solution. The EDC/NHS treatment increased the tensile strength and Young modulus values. In addition, the elongation at break parameter was lower than that of the pure collagen sample used as a control, which remains similar to the results of our study [33]. In a study performed by Nashchekina et al., the modification by EDC/NHS resulted in a reduction in films’ elasticity. This reduction was proportional to the concentration of the crosslinking agent used: as the crosslinker concentration increased, the films became less elastic [34]. The samples achieved maximum swelling degree approximately two hours after immersion in the PBS solution. The Coll_EDC film disintegrated the fastest, while the sample with NHS addition showed the highest degree of swelling. The immersion-crosslinked samples exhibited a significantly lower degree of swelling and were highly durable. Moreover, specimens crosslinked only with EDC showed a lower swelling degree compared to samples with the addition of NHS. Studies of EDC/NHS-crosslinked collagen membranes also demonstrated a lower rate of degradation of modified samples compared to non-crosslinked ones [31]. However, Nair et al. revealed that, as a result of their swelling analysis, it was observed that treatments did not significantly affect the material [27]. The crosslinkers modified collagen film surface morphology; the highest roughness was observed for collagen films crosslinked with EDC, whereas the lowest for specimens crosslinked with EDC with NHS addition. Crosslinking agents increased the surface roughness of collagen films, except for the samples modified with the addition of EDC and NHS mixture. According to Grover et al., EDAC/NHS crosslinking also decreased the roughness of collagen film [32]. Additionally, after crosslinking collagen-based materials with EDC/NHS, Zhang et al. obtained a sample with increased porosity [31]. All films were characterized by hydrophilic character. The films’ modification resulted in a decrease in their hydrophilicity and wettability and a decrease in the polar surface energy component. Water contact angle measurements conducted by Safandowska et al. also demonstrated that for collagen films crosslinked with EDC and NHS, the water contact angle values increased in comparison to native collagen film, and thus a decrease in the hydrophilicity of the obtained material was observed [35]. However, there were studies in which no significant differences in values of contact angle between pure collagen and crosslinked specimens were observed [31]. Conclusions obtained from the performed experiments expanded the state of research on the physicochemical properties of EDC/NHS-crosslinked fish collagen films in various conditions, which has the potential for use in biomedical applications. Our study shows that the manner in which the crosslinking is carried out is of great importance. The immersion method proved to be more effective. Samples crosslinked in this way were more durable and mechanically stronger. The use of EDC alone or coupled to NHS is also important. EDC helps to form the amide bond between the carboxyl and amino groups of the amino acids that make up collagen but does not become part of the crosslinked material. NHS aids in the action of EDC by increasing the stability of carbodiimide-crosslinked products by forming more stable esters. Therefore, it is a good solution for preparing biocompatible and stable collagen-based materials; however, in the case of biomaterials, it is necessary to conduct more advanced studies, including biological ones.

## 4. Materials and Methods

### 4.1. Preparation of Collagen Films

A total of 1% of collagen solution was prepared by dissolving freeze-dried collagen obtained from silver carp fish skin (supplied by SanColl Sp. z o. o., Rzeszów, Poland) in 0.1 M acetic acid (Stanlab; Lublin, Poland). The prepared solution was shaken and then stirred with a magnetic stirrer (500 rpm) at room temperature until the collagen was completely dissolved. The dissolution process took several days. 

Three 30 g portions of collagen were poured onto square polystyrene plates (10 cm × 10 cm). One was used as a control (*Coll*) and the other two were left for further modification by dipping in the solution of crosslinking reagents. Two portions of 30 g aliquots of collagen were also weighed and placed on the magnetic stirrer; to one portion, 120 mg of EDC (1-(3-dimethylaminopropyl)-3-ethylcarbodiimide hydrochloride; AmBeed, USA) (*Coll_EDC*) was added, and to the other portion, 12 mg of EDC and 18 mg of NHS (N-hydroxysuccinimide; TCI, Tokyo Chemical Industry, Tokyo, Japan) (*Coll_EDC/NHS*) were added. The samples were stirred for 15 min (500 rpm) at room temperature, and then poured onto polystyrene plates. All films were obtained by the solvent casting method at room temperature and the same conditions of humidity. Samples were dried for 5 days on a level surface.

Two water solutions were prepared for immersion crosslinking process: 50 mM EDC (*Coll_EDC_d*) and 50 mM/25 mM EDC/NHS (*Coll_EDC/NHS_d*). Pure collagen films were immersed in the prepared solutions (50 mL) for 24 h. After this period, the films were placed in 50 mL of PBS solution (prepared from PBS Tablets; Life Technologies Limited (Renfrew, UK)) and rinsed for 2 h; then, they were transferred to 50 mL of distilled water, rinsed for 1 h, and subsequently left to dry. The images of the films received are summarizsed in Table 6. 

### 4.2. Fourier Transform Infrared Spectroscopy

The chemical structure of the pure collagen films and modified films was evaluated by FTIR spectroscopy. Spectra were recorded using Nicolet iS10 equipment with a diamond ATR prism crystal accessory (Thermo Fisher Scientific, Waltham, MA, USA). The spectra were recorded in absorption mode in the wavenumber range from 400 to 4000 cm^−1^, with 4 cm^−1^ resolution using 64 scans. OMNIC 9.3.30 software was applied to process the data. 

### 4.3. Mechanical Testing

Mechanical properties were examined using a mechanical testing machine (Z.05, Zwick and Roell, Ulm, Germany). Evaluated parameters were as follows: Young modulus (GPa), tensile strength (MPa), breaking force (N) and elongation at break (%). The speed starting position was 50 mm/min, the speed of the initial force was 5 mm/min, and the initial force was 0.1 MPa. Samples were cut in the shape of paddles (with a width of 4 mm in the center) and up to ten samples of one type were examined. The TestXpert II 2017 program was used to collect the data, the results were presented as average values with standard deviation and Q-Dixon test was performed.

### 4.4. Swelling and Degradation Properties

Obtained films were cut into squares of similar weight and placed in the containers with 50 mL of phosphate-buffered saline (PBS) at 37 °C. Sample weights were measured at 0.5 h, 1 h, 2 h, 4 h, 8 h, 24 h, 48 h and 72 h. The swelling degrees were calculated using the following equation:

$$swelling=\frac{{(m}_{t\;}-m_{0\;})}{m_{0}}\;\times \;100\%\;[\%]$$

*m_t_*—Weight of the material after immersion in PBS [g]; 

*m*_0_—The initial weight of the material [g].

### 4.5. Atomic Force Microscopy

The microstructure of the collagen film surface was analyzed with images using an atomic force microscope from MultiMode Scanning Probe Microscope Nanoscope IIIa (Digital Instruments Veeco Metrology Group, Santa Barbara, CA, USA) with tapping mode at room temperature. The roughness parameters were calculated using Gwyddion 2.63 software.

### 4.6. Contact Angle and Surface Energy

Contact angle measurements were performed using a goniometer equipped with drop shape analysis (DSA 10 produced by Krüss, Hamburg, Germany). All measurements were carried out at room temperature using glycerin (G) and diiodomethane (D). The result of the contact angle for each film sample is an average value of up to ten measurements of individual droplets. The Owens–Wendt method was applied to calculate the surface free energy.

## 5. Conclusions

EDC and NHS are promising crosslinking agents for fish collagen modification. These compounds can be added to the collagen solution before casting or can be used as solution for collagen films to soak them. Both crosslinking conditions modified the surface roughness of collagen films and led to a decrease in their hydrophilicity and wettability. 

Our study shows that the manner in which the crosslinking is carried out is of great importance. The immersion method proved to be more effective. Samples crosslinked in this way were more durable, mechanically stronger and exhibited a significantly lower degree of swelling. Modified fish collagen can be used for biomedical and cosmetic applications.

Following the scientific reports, our research concerned the use of collagen derived from fish skins, which are usually constituted as a waste product of fish processing, and our results are in line with the current direction of development of studies on biopolymers. Our results regarding EDC/NHS crosslinking of fish collagen thin films may constitute the basis for further investigations and the creation of materials with biomedical and cosmetic potential. Future prospects for such further studies relate in particular to safety testing and collagen product stability. Some predictions about the stability of the films during the swelling and degradation tests are included in this research. However, more practical tests can be carried out in the future, particularly with regard to skin conditions. The presented method of crosslinking is rather uncomplicated and the obtained results allow for the further expansion of knowledge about the crosslinking of collagen films and their physicochemical properties. 

## Figures and Tables

**Table 1:** Band positions in IR spectra of native collagen film and films modified by EDC or EDC/NHS.

Sample	Band Position [cm^−1^]							
	Amide A	Amide B	CH_2_ Asymmetric Stretch	Amide I	Amide II	CH_2_ Bend	COO^−^ Symmetric Stretch	Amide III
Coll	3293	3071	2933	1629	1541	1450	1386	1235
Coll_EDC	3308	3068	2935	1632	1548	1448	1378	1237
Coll_EDC/NHS	3302	3070	2935	1629	1544	1451	1379	1233
Coll_EDC_d	3291	3072	2935	1629	1543	1450	1386	1236
Coll_EDC/NHS_d	3291	3074	2935	1629	1541	1451	1387	1235

**Table 2:** Swelling degree of collagen films non-crosslinked and crosslinked with EDC or EDC/NHS.

Sample	Time [h]							
	0.5	1	2	4	8	24	48	72
Coll	498 ± 18	663 ± 17	889 ± 38	800 ± 15	-	-	-	-
Coll_EDC	426 ± 24	498 ± 13	685 ± 38	-	-	-	-	-
Coll_EDC/NHS	782 ± 113	1019 ± 136	1045 ± 92	945 ± 151	-	-	-	-
Coll_EDC_d	146 ± 29	135 ± 32	132 ± 33	146 ± 24	119 ± 39	133 ± 31	148 ± 26	131 ± 31
Coll_EDC/NHS_d	181 ± 46	195 ± 31	190 ± 19	191 ± 40	195 ± 32	198 ± 32	173 ± 51	128 ± 92

**Table 3:** Rq and Ra values for the pure collagen film and collagen films modified with EDC and NHS.

	Rq [nm]	Ra [nm]
Coll	16.79 ± 0.50	13.57 ± 0.75
Coll_EDC	58.32 ± 4.97	46.79 ± 3.71
Coll_EDC/NHS	13.31 ± 0.42	10.70 ± 0.16
Coll_EDC_d	19.80 ± 2.04	15.75 ± 1.89
Coll_EDC/NHS_d	40.72 ± 6.29	33.01 ± 5.02

**Table 4:** AFM images of the surface of collagen films with EDC and NHS.

Coll 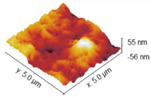	
Coll_EDC 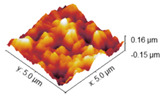	Coll_EDC/NHS 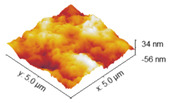
Coll_EDC_d 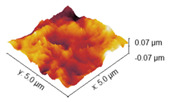	Coll_EDC/NHS_d 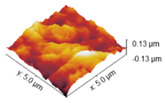

**Table 5:** Contact angle results for native collagen films and films crosslinked with EDC or EDC/NHS.

Sample	Θ Glycerine [°]	Θ Diodomethane [°]	IFT (s) [mJ/m^2^]	IFT (s, D) [mJ/m^2^]	IFT (s, P) [mJ/m^2^]
Coll	70.8	58.9	62.85	15.12	47.73
Coll_EDC	87.7	52.9	32.18	31.00	1.17
Coll_EDC/NHS	73.7	60.7	30.7	22.49	8.08
Coll_EDC_d	78.3	58.2	30.28	25.13	5.15
Coll_EDC/NHS_d	75.9	-	-	-	-

**Table 6:** Photos of all obtained collagen films on a blue background.

Coll	Coll_EDC	Coll_EDC/NHS	Coll_EDC_d	Coll_EDC/NHS_d
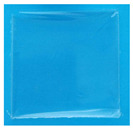	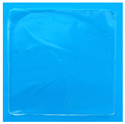	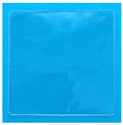	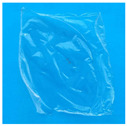	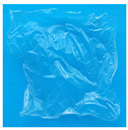

## Data Availability

Data protected by the company.
